# Supplementary material for: COZOID: contact zone identifier for visual analysis of protein-protein interactions
Source: BMC Bioinformatics. 2018 Apr 6;19:125. doi: 10.1186/s12859-018-2113-6 (PMC5889581; doi:10.1186/s12859-018-2113-6)
Supplement: Supplementary file 2 — Software build. Executable binary file of the software tool. (ZIP 118,784 kb) [file 12859_2018_2113_MOESM2_ESM.zip › cozoid/java/docs/beansbinding-1.2.1-doc.zip!/org/jdesktop/el/impl/parser/package-frame.html]

org.jdesktop.el.impl.parser (Beans Binding)


org.jdesktop.el.impl.parser

Interfaces 
  
*ELParserConstants*
  
*ELParserTreeConstants*
  
*Node*
  
*NodeVisitor*

Classes 
  
ArithmeticNode
  
AstAnd
  
AstBracketSuffix
  
AstChoice
  
AstCompositeExpression
  
AstDeferredExpression
  
AstDiv
  
AstDotSuffix
  
AstDynamicExpression
  
AstEmpty
  
AstEqual
  
AstFalse
  
AstFloatingPoint
  
AstFunction
  
AstGreaterThan
  
AstGreaterThanEqual
  
AstIdentifier
  
AstInteger
  
AstLessThan
  
AstLessThanEqual
  
AstLiteralExpression
  
AstMinus
  
AstMod
  
AstMult
  
AstNegative
  
AstNot
  
AstNotEqual
  
AstNull
  
AstOr
  
AstPlus
  
AstString
  
AstTrue
  
AstValue
  
AstValue.Target
  
BooleanNode
  
ELParser
  
ELParserTokenManager
  
SimpleCharStream
  
SimpleNode
  
Token

Exceptions 
  
ParseException

Errors 
  
TokenMgrError
